# Changes in Social Networks as a Response to Loneliness Among Older Adults

**DOI:** 10.1093/geroni/igaa057.1930

**Published:** 2020-12-16

**Authors:** Abhijit Visaria, Pildoo Sung, Angelique W M Chan

**Affiliations:** Duke-NUS Medical School, Singapore, Singapore

## Abstract

The expression of loneliness suggests perceived social isolation and the lack of meaningful, rewarding and desired social connections. In this paper we seek to estimate whether loneliness among older adults is related to subsequent changes in two attributes of their social networks, i.e. the size and frequency of contact with network members. We use the Panel on Health and Ageing of Singaporean Elderly, a nationally representative study of older adults aged 60 years and older, conducted in 2009 (N=4990) with two follow-up waves in 2011 and 2015, and measure loneliness in terms of relational connectedness, i.e. feeling a lack of companionship, and social connectedness, i.e. feeling left out. Our preliminary analysis shows that loneliness is associated with a subsequent reduction in the size but not strength of social networks. We further propose to assess the causal relationship using cross-lagged panel analysis, thereby accounting for the possibility of a reciprocal relationship.

